# Investigating US medical students' motivation to respond to lapses in professionalism

**DOI:** 10.1111/medu.13617

**Published:** 2018-06-25

**Authors:** Marianne Mak‐van der Vossen, Arianne Teherani, Walther N K A van Mook, Gerda Croiset, Rashmi A Kusurkar

**Affiliations:** ^1^ Department of Research in Education VUmc School of Medical Sciences Amsterdam University Medical Centers Amsterdam the Netherlands; ^2^ LEARN! Research Institute for Education and Learning VU University Amsterdam the Netherlands; ^3^ Center for Faculty as Educators School of Medicine University of California San Francisco San Francisco California USA; ^4^ Department of Intensive Care Medicine Maastricht University Medical Centre Maastricht the Netherlands; ^5^ Faculty of Medical Sciences University Medical Center Groningen Groningen the Netherlands

## Abstract

**Context:**

As unprofessional behaviour in physicians can compromise patient safety, all physicians should be willing and able to respond to lapses in professionalism. Although students endorse an obligation to respond to lapses, they experience difficulties in doing so. If medical educators knew how students respond and why they choose certain responses, they could support students in responding appropriately.

**Objectives:**

The aim of this study was to describe medical students' responses to professionalism lapses in peers and faculty staff, and to understand students' motivation for responding or not responding.

**Methods:**

We conducted an explorative, qualitative study using template analysis, in which three researchers independently coded transcripts of semi‐structured, face‐to‐face interviews. We purposefully sampled 18 student representatives convening at a medical education conference. Preliminary open coding of a data subset yielded an initial template, which was applied to further data and modified as necessary. All transcripts were coded using the final template. Finally, three sensitising concepts from the *Expectancy–Value–Cost* model were used to map participants' responses.

**Results:**

Students mentioned having observed lapses in professionalism in both faculty staff and peers. Students' responses to these lapses were avoiding, addressing, reporting or initiating policy change. Generally, students were not motivated to respond if they did not know how to respond, if they believed responding was futile and if they feared retaliation. Students were motivated to respond if they were personally affected, if they perceived the individual as approachable and if they thought that the whole group of students could benefit from their actions. *Expectancy of success*,* value* and *costs* each appeared to be influenced by (inter)personal and system factors.

**Conclusions:**

The *Expectancy–Value–Cost* model effectively explains students' motivation for responding to lapses. The (inter)personal and system factors influencing students' motivation to respond are modifiable and can be used by medical educators to enhance students' motivation to respond to lapses in professionalism observed in medical school.

## Abstract 2

While students can be reticent to respond to professionalism lapses, this research identifies modifiable system‐related features that might help.

## Introduction

Approximately 60% of medical students observe lapses in professionalism by faculty staff and peers in medical school.^1^ Each year up to 19% of medical students fail a professional behaviour assessment.^2^, ^3^, ^4^, ^5^ Although all physicians should be willing and able to respond to professionalism lapses in colleagues,^6^ it is not always easy to do so. For medical students, who are still learning and are dependent on their teachers for grades, it is particularly difficult. Although medical students endorse a professional obligation to respond to professionalism lapses,^7^ they experience difficulties in meeting this obligation.^8^ It is still unclear what motivates students to overcome these difficulties and how they actually respond. Knowledge about students' motivation to respond will allow educators to support students in responding to observed professionalism lapses.

Medical professionalism can be defined in many ways.^9^ The essence of these various definitions refers to the necessity for physicians to adhere to high ethical and moral standards in order to gain the trust of their patients. Professionalism lapses can be defined as instances in which physicians fail to gain the trust of patients or colleagues, or faculty members fail to gain the trust of students or colleagues, or students fail to gain the trust of teachers or peers. Lapses, by either students or faculty staff, are occasionally serious, such as when medical records are falsified or sexual harassment occurs, but are more often less egregious, such as when an individual displays a lack of engagement, lack of respect or lack of insight into his or her own behaviour.^6^, ^10^, ^11^, ^12^ Displaying a lapse in professionalism does not automatically indicate that the individual is an ‘unprofessional’ person: many professionalism lapses result from poorly navigated responses to interpersonal and system factors in the workplace, to which we are all vulnerable.^13^ However, even less egregious lapses can have adverse effects. Recently, Cooper et al.^14^ reported that unsolicited patient observations of unprofessional behaviours in a surgeon (e.g. relating to disrespectful communication or poor availability to patients) were associated with complications for the surgeon's patients. Thus, in acknowledgement of the relevance of unprofessional behaviours to patient safety, physicians should respond to such behaviours and openly discuss them. The goal of this is to learn from lapses and ultimately to influence personal, interpersonal and system factors to prevent future lapses.^6^

Although medical educators feel highly responsible for the teaching and learning of professionalism in medical school, they do not always report professionalism lapses by students.^15^ Recent research reveals several personal and institutional barriers that explain why teachers remain silent when witnessing lapses.^16^ Although these barriers may be understandable, by doing this faculty staff nevertheless end up role‐modelling to their students that it is not worth responding to lapses of professionalism. Recommended responses for medical students who observe professionalism lapses are: ignore it; challenge the individual; discuss the lapse with peers, or report the lapse to a faculty member.^12^ Regardless of these, it is not clear *how* medical students respond and *why* they choose a particular way of responding. It is clear that students are reluctant to report professionalism lapses to a higher authority.^17^, ^18^, ^19^ We also know that students experience difficulties in challenging an individual after observing a morally troubling situation. These difficulties arise from personal and systemic constraints.^20^, ^21^ Personal constraints include a lack of confidence in one's own knowledge and judgement; systemic constraints include fear of repercussions for grades or opportunities, fear of damaging relations, and hierarchy.^20^

The *Expectancy–Value–Cost* model of motivation, an update of Eccles et al.'s *Expectancy–Value* model, can help to elucidate students' choices of how and why to engage in responding to professionalism lapses that students observe in faculty staff or in peer students.^22^, ^23^ The model describes a person's motivation to engage or not engage in a certain task as being based on the balance of the *expectancy* of being successful in that task (*Can I do it?*), the perceived *value* of engaging in the task (*Do I want to do it?*) and the *costs* of engaging in the task (*Are there barriers that prevent me from doing it?*). The model divides *value* into three qualities: intrinsic value (enjoyment); extrinsic value (usefulness and the ethical values of socialising agents like teachers), and attainment value (individual identity factors like relatedness, competence and esteem).

This exploratory study investigated how medical students respond to unprofessional behaviours they observe in peers and faculty staff, and what motivates them to choose a certain response. In addition, we explored how the teaching of responses to professionalism lapses, based on students' propositions, can be incorporated into a medical curriculum.

## Methods

We designed an explorative, qualitative interview study using thematic analysis to capture the experiences of the participating medical students.^24^ The study was set up using a constructivist paradigm, in which data and analysis are created based on the interaction of the experiences of both participants and researchers.^25^ In acknowledgement of the influence of the researchers, we share the following information with the reader: all of the present authors are educational researchers and/or medical educators experienced in the teaching and guidance of professionalism in medical students. MM‐vdV, WNKvM, GC and RAK are medical doctors.

### Setting and participants

We interviewed students at a medical education conference during which representatives from all sectors of US medical schools and teaching hospitals convened to discuss the future of academic medicine. To gather a variety of experiences from different settings, we created a purposeful sample of second‐, third‐ and fourth‐year students representing different medical schools by reaching out to the organisation of student representatives. We aimed for at least 15 participants. We did not sample first‐year students because they may not yet have had the experiences this study aimed to explore. We specifically sampled student representatives because, by taking on the role of a representative, they show themselves to feel responsible for the quality of teaching and learning in their medical school. We also expected them to have a broader understanding of institutional policies and procedures than most medical students. We expected that interviewing these proactive students would yield a wide range of responses to professionalism lapses and assumed that these responses might also be noticeable in the wider student body.

### Interviews

We conducted semi‐structured, face‐to‐face interviews with the participants, lasting approximately 30 minutes each, using an interview scheme based on the literature and our personal experiences. Interviews began with the question: (i) What does your institution expect from you regarding professionalism and do you align with that? Participants were then asked to recall a situation in which they had observed a professionalism lapse in a peer or in a faculty member (teacher, resident, attending doctor). Subsequent questions were: (ii) How and why did you respond to the observed professionalism lapse of a peer student? (iii) How and why did you respond to the professionalism lapse of a faculty member? (iv) Which alterations in the curriculum do you propose to medical educators to make it easier for students to respond to professionalism lapses?

### Procedure

MM‐vdV invited student representatives to participate. Before each interview, the participant was informed about the research protocol and assured that the interview was completely voluntary and that all data would be handled anonymously to ensure confidentiality in all circumstances, after which consent was obtained. Participants received a US$15 gift card for their participation. MM‐vdV or a trained research assistant, neither of whom were related to the student's school, conducted the interviews. All interviews were audiorecorded and transcribed verbatim.

### Data analysis

We used atlas.ti (Scientific Software Development GmbH, Berlin, Germany) to organise the coding. Data were coded in three consecutive steps. The first step consisted of independent open coding of two transcripts by three of the investigators (MM‐vdV, AT, RAK). They reached consensus about an initial set of codes and themes. MM‐vdV used this initial set to code all transcripts, discussed any difficulties with the other two coders, and thus generated a thematic map of the analysis (Appendix S1). MM‐vdV used this final map of codes and themes to code all transcripts again.^24^ The last step also included the use of *sensitising concepts*.^26^ Sensitising concepts are general ideas that suggest different directions from which to see, organise and understand the experiences of participants. In a discussion among the three coders, participants' answers to interview questions 2 and 3 were mapped to the sensitising concepts *expectancy*,* value* and *costs* from the *Expectancy–Value–Cost* model of motivation.^22^

This study was qualified as exempt from requirements for ethical approval by the University of California, San Francisco Institutional Review Board.

## Results

**Figure 1:**
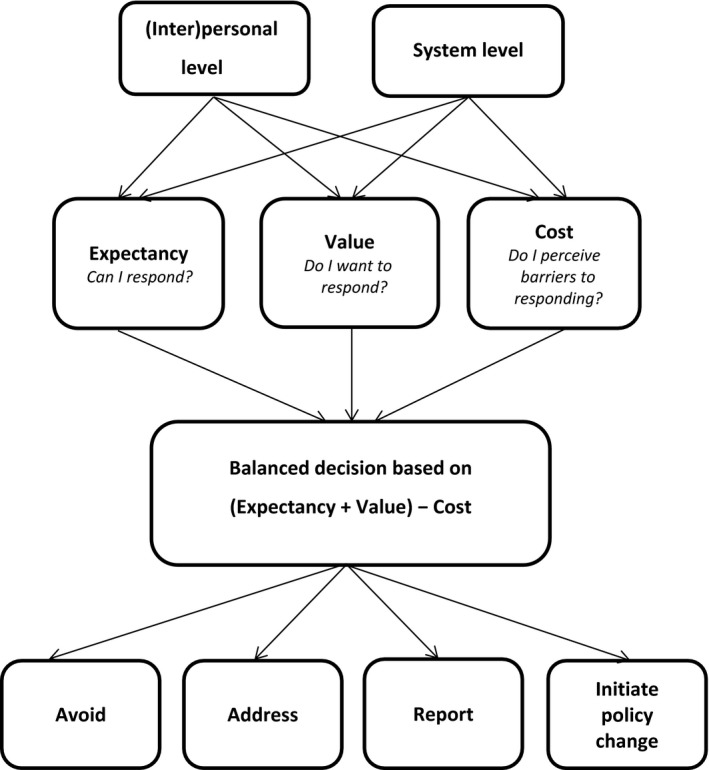
Expectancy, value and costs influencing students' responses to lapses of professionalism in peers and faculty staff

We interviewed 18 student representatives (10 female, eight male) from 17 different US medical schools (12 public, five private). Eight participants were in year 2, four in year 3 and six in year 4 of medical school. Interviews lasted between 25 and 40 minutes. Students had responded to an observed professionalism lapse by a faculty member or peer student by *avoiding*,* addressing* or *reporting* the lapse, and/or by *initiating policy change*. The balance of *expectancy of success*,* value* and *costs*, each influenced by factors on personal and system levels, determined which response was chosen (Fig. 1).

### Students' alignment with their school's definition of professionalism

Most students were able to cite their medical school's definition of professionalism, and all students knew of its existence and where to find it. Students' own perceptions of professionalism did generally align with their school's definition, although they sometimes disagreed with the way the school operationalised the professionalism values into attendance rules:

How we do ‘attendance’ factors into our grade. A lot of people don't like to go to lecture[s], but [would] rather go to a study room and study. That is deemed unprofessional behaviour. I think sometimes these policies, although good‐natured, can cause people to think professionalism in the school is a joke. (Student 6)

Alignment with the school's definition of professionalism was more common for students who felt that they had a voice in the formulation of the school's professionalism code.

Students write their own honour code at the beginning of medical school. Every person in the class signs the code. It's framed and it's hung up in our lecture hall to remind us that these are the behaviours that we expect students to have. (Student 7)

In addition, even if they agreed with their school's definition of professionalism, students found that measures taken against students who displayed unprofessional behaviour were sometimes too strict:

I think that, because some professionalism studies have correlated students being late to later issues in professionalism, I think that they kind of grab on to that notion and run with it and perhaps are a little bit too harsh in certain instances where, you know, so a student is late a couple times, it maybe isn't such a huge issue. (Student 3)

By contrast, students appreciated a prompt response by faculty staff and their addressing of unprofessional behaviour in order to remediate it:

I think my perception is that they address it rather soon, so as soon as they notice that maybe somebody is not behaving the way that the school's mission statement aligns with, they'll meet with them rather than trying to ruin their future. You know what I mean? Even if they did something really, really bad, they just try to address it right away. (Student 5)

### Character of responses

All students had observed professionalism lapses in peers, as well as in faculty members. The types of behaviours did not differ between these two groups and could be themed as indicating problems with *involvement*,* integrity*,* interactions*, and *insight*. Table 1 shows examples of professionalism lapses as observed and described by the participants.

**Table 1:** Examples of lapses of professionalism as described by participants, categorised into four themes

Theme	Examples of professionalism lapses in faculty staff	Examples of professionalism lapses in students
Involvement	Faculty member does not respond to students' e‐mails	Student has issues regarding timeliness
	Professor is not prepared for lecture	Student lets others do the extra work
Integrity	Resident never uses the hand sanitiser or washes hands when going from room to room	Student is very competitive, taking credit for other students' work
	School is not accountable for administrative mistake with lottery system for placements for clerkships, even denying that there was a problem	Student copies notes from others, which is not allowed
Interaction	Surgeon calls the patient names and stuff in the operating room	Student displays disrespectful behaviour to another student about gender issues, politics and religion
	Attending doctor is texting and calling the student in a really inappropriate way	Student posts a message on social media that is derogatory to a professor
Insight	Educator is too personal, making jokes about the medical procedures he's having that week	Student is selling nutritional supplements, suggesting that he is an expert
	Faculty member puts forward a strong own opinion, and is not open to different opinions within small student groups	Student becomes abrasive and dismissive of others who have very good ideas, but cannot express it because he believes that it is his view that works and no‐one can convince him otherwise

The types of responses to these lapses did not differ between the two groups. Students responded to professionalism lapses by both peers and faculty staff in four different ways: *avoiding*;* addressing*;* reporting*, and/or *initiating policy change*. Table 2 gives a description of each response and sample quotes for each of the options.

**Table 2:** Sample quotes for responses to professionalism lapses of peers and faculty

Theme	Responses to lapses of peers	Responses to lapses of faculty
*Avoid* Acknowledging the lapse, but not taking any action	*I thought about it a lot because I think the thing that they wanted me to do was to laugh and I didn't feel comfortable laughing with them, but unfortunately, I didn't say anything either. I was just kind of silent*. (Student 10)	*I smiled and I was like, oh, that's good. I just moved on, because I really didn't know what to say. I had to be with him for the rest of the day. I didn't know who to complain to, I didn't know if I could change physicians. I felt a little stuck*. (Student 9)
*Address* Discussing the lapse with the observed person.	*I texted her and said “*Hey, you may not want to post that here, it seems like it's a little bit too far*,” and she did took it down probably about ten minutes after it was posted*. (Student 3)	*We arranged a meeting with the Professor where we discussed his opinion and how our opinion differed and how we felt about what he had said*. (Student 3)
*Report* Informing a higher authority about the lapse.	*We don't tell names, but we tell the administration*. (Student 6)	*I had never written that a Professor should not work with students, and this was the first time I had done that, knowing that he would know who wrote that*. (Student 7)
*Initiate policy change* Changing system factors to prevent similar lapses in the future.	*Seems a little bit harsh, (i.e. students receiving an unprofessional behaviour judgment for not scheduling their exam in time) so the conversation that we had with administration, I was on student government, was to change this from a punitive thing, like you would get a demerit of sorts, into just some very strong, “*We advise you very strongly to schedule this by this time for these reasons.*”* (Student 1)	*I am part of a group that does report to faculty on issues like that (i.e. biased statements of faculty) and basically when we see something like that come up we just take note of it and report it to the faculty*. (Student 4)

#### Avoiding

Avoiding the unprofessional behaviour did not always mean that the student did not respond at all; an example of an avoidant response was that the observer became less likely to help the perpetrator:

I think once I start to perceive this what I thought was unprofessional competitive behaviour, that made me less likely to help this person. (Student 1)

#### Addressing

When students decided to address the unprofessional behaviour, they sometimes responded in the moment by, for example, making a joke, posing a question or addressing the behaviour directly. They might also respond after the moment by conducting a strategic discussion with the perpetrator. A personal reflection on how to react appropriately or a discussion among peers to verify their own perceptions always preceded this response. Peer discussions sometimes resulted in collective interventions to address the unprofessional behaviour, such as in a group discussion with the perpetrator. In such interventions with faculty staff, students muted their voice as they tried to de‐escalate the lapse as much as possible:

It was more muted when we were talking to the professor, of course, because we didn't want to come off as unprofessional. (Student 3)

Notably, students also mentioned defending peers when teachers asked for information about a peer's behaviour, even when they found the behaviour was not appropriate:

This is a tricky thing, because when the attendings or the residents would ask me where this person was, I didn't want to get them in trouble, so I wouldn't say: ‘Oh they left.’ I would say: ‘I'm not sure where they are.’ (Student 12)

#### Reporting

Concerns were very occasionally relayed to a higher authority and only when this was deemed to be absolutely required, such as in the case of behaviour that would affect patients in a negative way. If students decided to report a lapse, they favoured reporting to a student council over reporting to the clerkship director or dean. Reporting to a higher authority was preferably done anonymously, although students acknowledged that authorities could not take action on anonymous complaints. Although students were reluctant to report the lapses of faculty members to higher authorities, they regularly mentioned observed lapses in anonymous course evaluations.

#### Initiating a policy change

Participants took action as student representatives by making the problem visible to their peer students and responsible faculty staff with the aim of initiating a policy change:

As a representative, I hope I kind of set an example almost for my school and my classmates and just as a representative for just our class in general. Just kind of standing up, saying: ‘Hey, it's okay that these, that things happen. It's not okay that it did happen, but there are ways to move forward.’ (Student 15)

I'm also close with most of the deans. If someone were to approach me, I would feel comfortable talking to the deans. (Student 14)

### Motivation to respond to professionalism lapses

We were able to map all codes coming from interview questions 2 and 3 to the sensitising concepts *expectancy*,* value* and *costs*.

#### Expectancy of success

Expectations of success in responding to professionalism lapses in others appeared to be dependent on (inter)personal and system factors. Addressing was expected to be successful if the student saw him‐ or herself as assertive, if a good relationship had already been established with the observed person, and if a feedback‐giving culture existed in the medical school:

You know, I've never, like I said, been formally instructed on what the appropriate [way] is to give feedback in a professional environment, but I think I myself, I would feel I would be assertive enough to just say: ‘Hey, I noticed that this happened. It made me feel uncomfortable.’ (Student 14)

I knew him from before, so I felt like I could tell him that. (Student 5)

Our school has kind of set a tone that we give a lot of feedback to our lecturers, we get a lot of feedback from lecturers; individual feedback on how we perform in small groups and we give a lot of feedback to our peers and it's required that we give this feedback, so I think we're just kind of now in a culture where we expect people to tell us what we're doing. (Student 10)

Addressing was expected to be less successful, and thus avoided, if the observed person was angry, not approachable, or defensive in his or her reactions:

If they have really aggressive personalities, very antagonising behaviours, I won't say anything about the unprofessional behaviour. (Student 5)

Students indicated that they found communication about professionalism lapses difficult and that they did not know how to respond effectively. Reporting was hampered by a lack of knowledge about the reporting system. Addressing was hampered by a lack of specific skills to communicate in difficult circumstances:

How do you bring it up in a way that you don't hurt their feelings or don't get them in trouble, but, at the same time, have them stop that unprofessional behaviour? (Student 12)

An existing hierarchy between the student and perpetrator made this more difficult:

We kind of felt, as the students, there were two students, and then it was just all these residents and the attending. We felt very uncomfortable and very outnumbered. (Student 12)

#### Value

Value (a higher value increases the likeliness of responding to unprofessional behaviour) also appeared to be dependent on (inter)personal and system factors. Interpersonal and personal factors were described as feelings of responsibility for their own education and the education of other students:

Because I think it's important that we kind of share and help build each other up and make sure that we also are letting each other know what our weaknesses are. (Student 15)

System factors were described as feelings of responsibility for the well‐being of patients, or the reputation of the profession as a whole:

I guess ultimately the standard that I hold is when does the so‐called lack of professionalism actually affect the care the patient has. (Student 1)

Because I think at the end of the day there's a lot of unprofessional behaviour towards medical students, and that's one thing, I think I can handle people mistreating me, but when I feel that a patient is being impacted… (Student 16)

Trainees should have the ability to communicate among themselves, because we're going to be communicating with colleagues and people above us for the rest of our lives. So, we need to be able… That needs to start being ingrained within our conduct. So it… We need to be able to openly talk about anything. Even things that are [of] conflict. (Student 11)

Students expressed the idea that they, during their medical education, had built up a tolerance for unprofessionalism and thus sometimes perceived responding to unprofessionalism as futile:

Maybe I just have a tolerance for unprofessionalism now. (Student 1)

I think it was mainly feelings of futility that prevented me from going to the dean. (Student 8)

#### Costs

High costs made responding to professionalism lapses less likely. Costs were also contingent on (inter)personal and system factors. The idea or action of responding to unprofessional behaviour made students nervous. Students did not want to be seen as troublemakers, whiners or tattletales:

You don't want [the] attending to think that you're ‘difficult’ and ‘hard to work with’. (Student 5)

As such, students worried that relationships could be damaged. Students feared personal retaliation that might affect their academic grades, their education and their future:

We don't report anything because we're too afraid for negative implications for our future career. (Student 5)

The costs of responding to behaviours in peers were perceived to be lower than those of responding to behaviours in faculty staff. Costs were also perceived to be lower in the case of a collective response:

I think as the peers were better at keeping people in, like, more in line because if someone does something that seems a little bit unprofessional, then you feel more comfortable approaching the peer about it than you do a teacher. (Student 10)

We kind of both did [it] together and I think what kind of made it easier was that there were two of us. (Student 10)

### Students' recommendations

Students suggested changes in the curriculum to guide them in how to respond to lapses of professionalism in peers and faculty staff. They formulated options to strengthen awareness, knowledge and skills related to professionalism in students and in faculty staff, and also made recommendations for changing system aspects of the curriculum.

#### Strengthening of professionalism in students

Several options for strengthening professionalism in students were mentioned: showing students the link between unprofessional behaviour and patient safety; discussing the school's expectations, and offering practical sessions in which students learn how to address professionalism lapses in both equal and hierarchical situations. Students indicated that they would value having a credible and trustworthy mentor with whom to speak about professionalism dilemmas. This might be an older student, which would create support among students. This way, the school could provide students with a space in which to discuss their experiences without fear of repercussion:

I think that if you create a space where people can raise concerns without jeopardising… overall, balancing the concerns around jeopardising your social standing, your future peers' careers and your own career, which is a lot to balance, certainly. I think any work you take to mitigate some of those concerns, I think it makes students more likely to feel comfortable doing it. (Student 8)

#### Improvements for professionalism in faculty staff

Suggested improvements for professionalism in faculty staff included that faculty members model the right behaviours in a better way, including by taking responsibility for addressing unprofessional behaviour in a timely fashion. Students advocated that faculty members respond to lapses in a non‐punitive, pedagogical way with the intention of letting the student learn. Students suggested that faculty staff need to reserve punitive actions for students who fail to respond to this pedagogical approach:

I think if they modelled that behaviour for us, that will help us feel more comfortable also doing that. (Student 12)

With our clerkships and in their work here, they could try to make it more a part of our curriculum that we are working together, and we are working for the benefit of everyone in our team. We're not casting blame or undue responsibility. It would take a large structural change. (Student 4)

#### Change of system aspects

With respect to changes of system aspects, students suggested that institutions formulate their rules and regulations in collaboration with students, thus providing clarity to students about the values upon which professionalism evaluations are based. Participating student representatives were very clear that initiatives for suggested changes should preferably come from students themselves. Thus, they recommended deliberately involving students in policymaking at medical schools:

Who could change that are the people who do have the power to change policies and the students who can talk to the people who can change policies and provide them [with] their point of view and perspective, but the people who are in charge need to be willing to open up and listen to the students and their concerns about these issues first before they can even think of addressing these policies. (Student 6)

## Discussion

The aim of this study was to investigate medical students' responses to professionalism lapses observed in medical school, and their motivation to respond. In addition, we explored if students aligned with their institutions' definitions of professionalism, and what alterations in the curriculum they would propose to facilitate responding to professionalism lapses.

### Students' alignment with their schools' definitions of professionalism

Students broadly aligned with the professionalism values of their institution, although accountability was difficult to align with if it was merely translated into mandatory tasks or attendance. In their opinion, this does not reflect the goal of accountability as the self‐regulation of the professional community to ensure competent practice by physicians.^27^ Based on our findings, it seems that the translation of the professional value of accountability to rules or mandatory tasks can cause students to narrow their perceptions of accountability to a minimal effort (i.e. of simply showing up) and can diminish students' capacity to recognise and consider the broader concept. This indicates that the translation of professional values into rules and regulations in medical schools is not easy.^28^

### Responses to professionalism lapses

Our findings are based on experiences of medical students, by contrast with earlier findings that come from simulated circumstances and questionnaires, or from residents.^8^, ^29^ Roff and Dherwani investigated medical educators' advice for students to respond, which included *ignore*,* challenge the individual*,* discuss the lapse with peers* or *report*.^12^ Our findings resemble these recommendations, but we found that although students sometimes seem to ignore lapses, this does not always mean the student does nothing at all. We saw that after *avoiding* a lapse, all students, without exception, discussed the observed lapse with peers. These discussions helped them to decide on how to proceed individually or collectively. We confirm that students do indeed sometimes follow Roff and Dherwani's^12^ recommendation to challenge the individual, but they remain very reluctant to report the behaviour to a higher authority. An additional type of response that we found is *initiating policy change*: students, as representatives of the student body, thus acquire the power to influence the medical school. Through this influence, they try to change system factors that contribute to professionalism lapses. This is crucial because these student leaders are likely to be the future change agents the medical profession needs.

### Factors that influence motivation to respond

This study has uncovered several factors that relate to the motivation of students to respond to professionalism lapses in medical school. All factors could be mapped to the *Expectancy–Value–Cost* model.^22^ Our addition to this model is the distinction of (inter)personal and system factors for each of the three sensitising concepts *expectancy*,* value* and *costs*. We found some of the factors to be modifiable, which means that they could be used to design educational interventions to enhance students' motivation to respond to professionalism lapses.

#### Expectancy of success

This study reveals that students feel they are not always able to respond to professionalism lapses. Speaking about unprofessional behaviours is relatively underemphasised in medical curricula.^29^, ^30^ This factor seems to be highly modifiable: responding to unprofessional behaviours can be taught in medical school to provide students with the skills to do so.^31^ The expectancy of success is also higher if faculty members are approachable and the school has a feedback culture.

#### Value

We confirm Tucker et al.'s^8^ findings that students are more motivated to address lapses if there is a chance of harm to patients, which reflects the intrinsic value of feeling responsible for patients. We also found extrinsic value (e.g. ‘We have to do it as physicians so we must learn it now’) and attainment value: students were motivated to respond if their actions might lead to improvements for other students. Value factors were not the most important of the barriers we found, but could nevertheless be positively modified by providing students with the knowledge base of professionalism.^32^ In addition, professionalism values should preferably be discussed among teachers and students to obtain bidirectional alignment.

#### Costs

The most important costs, leading to avoiding to respond, were negative psychological experiences like anxiety, fear of failure or being uncomfortable. Students expressed their anxiety about experiencing retaliation, varying from retaliation that affected their grades to retaliation that caused them to miss out on teaching opportunities or career opportunities. Furthermore, fear of not fitting into the group and damaging relations were important costs. Like Kohn et al.,^33^ we found that directly addressing an individual is perceived as less costly than reporting. Costs can be mitigated by making the task of responding easier. This was the case when students felt support from the organisation, such as when there was a possibility of bringing their concern to a student council or a faculty member instead of acting themselves.

Our findings suggest that the factors that positively influence student motivation and that derive from the (inter)personal level (knowledge, skills, existent positive relations, own or other students' learning being affected) make the addressing of a lapse more likely. Motivational factors that derive from the system level (approachable faculty staff, feedback culture, strong professional values, organisational structures like a student council) appear to make the reporting of a lapse for a student easier. The condition for students to take action to make a policy change seems to depend on the combination of factors that foster motivation on both the (inter)personal level as well as the system level. Initiating a structural change in the curriculum or educational process requires personal leadership qualities, but also an institutional system that encourages student engagement and cultivates collective accountability.

### Students' recommendations

Students' propositions for alterations in the curriculum remarkably resemble some of the *new assumptions* described by Lucey and Souba: lapses are a part of learning; responses to these lapses should be pedagogical, and the community of practitioners must assume responsibility for supporting colleagues in remaining professional.^6^ Responding to patient safety issues has been promoted in recent decades, which has resulted in more willingness to respond to such issues.^34^ Similarly, responding to professionalism lapses needs to be given attention because the impact of unprofessional behaviour on patient outcomes has been proven.^14^, ^29^ How participants' recommendations can be implemented remains challenging and deserves further research. We know that educators' modelling of appropriate responses to inappropriate behaviours is crucial.^35^ Students' recommendations also confirm earlier advice that students need to be offered space in which to discuss their experiences with peers in sessions that will not be assessed.^36^

### Implications of the study findings

In this study, we were able to define modifiable factors that could enhance students' motivation to respond to professionalism lapses. Based on these factors, and the suggestions for improvement to the curriculum given by the participants in this study, we formulated recommendations for pedagogical and institutional strategies (Table 3).

**Table 3:** Pedagogical and institutional strategies to enhance students' motivation to respond to professionalism lapses

	Improving expectancy of success	Improving value of professionalism	Diminishing cost of responding
Pedagogical strategies	Teach practical skills how to address professionalism lapses Inform students about the routing when reporting professionalism lapses	Teach the cognitive base of professionalism Stress the effect of professionalism lapses on patient‐care Stress the effect of professionalism lapses on students' learning Create opportunities for students to interact with diverse patient groups	Stimulate critical responses of students by openly asking for it Evaluate professional behaviour formatively and timely Ensure that mistakes are openly discussed to create learning opportunities Offer room to students to discuss their experiences with peers in sessions that will not be assessed
Institutional strategies	Ensure that teachers are approachable Create a culture of feedback Make confidential ‘triage’ of observed professionalism lapses possible Facilitate small group teaching Centralize complaints management Make collective responding possible Inform reporting students about outcomes of investigations that took place after reporting of lapses	Set values in collaboration with students to create bidirectional alignment Make students part of policy making Ensure that teachers maintain the school's rules and function as role‐models who display professional behaviour Make reporting of professionalism lapses of faculty possible for students	Provide options for confidentially reporting Install a student council that is partly responsible for handling students' professionalism lapses

### Limitations of this study

Our decision to sample student representatives may explain why we found students willing to act upon professionalism lapses and to try to create changes in the curriculum. We chose to study student representatives based on the assumption that their responses would also be noticeable in the wider student body. Further research should reveal if this is the case, and if modifying the factors we found indeed enhances the motivation of all students to respond to professionalism lapses in medical school.

We asked the participants to talk about observed professionalism lapses and their responses to these. Theoretically, this implies that we did not find instances in which unprofessionalism was not registered at all (i.e. when the student did not consider the behaviour to be unprofessional). A subsequent question concerns whether others, such as patients or educators, would have different opinions. Furthermore, we spoke only to US students, which means that transferability to other cultural contexts may be limited and should be further investigated.

## Conclusions

Student representatives respond to lapses of professionalism they observe in faculty members or peer students by *avoiding*,* addressing* or *reporting* the lapse, and/or by *initiating policy change*. The balance of *expectancy of success*,* value* and *costs* determines which response is chosen. All three of *expectancy of success*,* value* and *costs* appear to be influenced by factors on the (inter)personal and system levels. Medical educators can use these factors to enhance students' motivation to respond to the lapses of professionalism they observe in medical school.

## Contributors

all authors contributed to the study design and to the analysis and interpretation of the data. MM‐vdV acquired the data and drafted the manuscript. AT, WNKvM, GC and RAK critically revised the manuscript for important intellectual content. All authors approved the final manuscript for publication and have agreed to be accountable for all aspects of the work in ensuring that questions related to the accuracy or integrity of any part of the work are appropriately investigated and resolved.

## Funding

none.

## Conflicts of interest

none.

## Ethical approval

this study was deemed to be exempt from requirements for ethical approval by the University of California, San Francisco Institutional Review Board.

## Supporting information


**Appendix S1.** Thematic map of the analysis.
