# Supplementary material for: Investigating US medical students' motivation to respond to lapses in professionalism
Source: Med Educ. 2018 Jun 25;52(8):838–50. doi: 10.1111/medu.13617 (PMC6055660; doi:10.1111/medu.13617)
Supplement: Supplementary file 1 — Appendix S1. Thematic map of the analysis. [file MEDU-52-838-s001.docx]

Code-Filter: All

______________________________________________________________________

HU: Student study coding

File: [\\vumc.nl\home$\store4ever\M.Mak\Documenten\Scientific Softwar...\Student study coding.hpr7]

Edited by: Super

Date/Time: 2017-03-02 16:08:13

______________________________________________________________________

# 1. Medical school definition

## 1.1 Do they know it

### 1.1.1no

### 1.1.2 yes

1.1.2.1 code of ethics of AMA

1.1.2.2 knows where to find it

1.1.2.3 several professionalism committees

1.1.2.4 document, and every syllabus speaks of professionalism

1.1.2.5 worksheets and pamphlets

1.1.2.6 professionalism workshops

1.1.2.7 student handbook

1.1.2.8 implicit teaching more important than explicit teaching

1.1.2.9 role modelling more important than formal education

1.1.2.10 professionalism rules also valid for faculty

1.1.2.11 students will not act professionally when they are not treated professionally

1.1.2.12 text book

1.1.2.13 code of conduct

## 1.2 Do they agree with it? Why

### 1.2.1 no

1.2.1.1 faculty use professionalism when it is not applicable

1.2.1.2 I do not have a voice in it

1.2.1.3 professionalism of institution too narrow, only focusing on unprofessional behavior, instead of professional behaviors on

1.2.1.4 students not involved in setting rules

1.2.1.5 this makes clear to me that you sometimes just have to align without thinking yourself

1.2.1.6 I do not agree with the institution, but for reasons of fairness all students have to follow the rules

1.2.1.7 too much focusing on attendance alone make students think that professionalism is a joke

### 1.2.2 yes

1.2.2.1 yes, students write their own honor code

1.2.2.2 social contract

1.2.2.3 start early

### 1.2.3 yes and no

1.2.3.1 I miss the aspect of justice

1.2.3.2 not agreeing with mandatory presence and rules to maintain that

1.2.3.3 school cracks down quite harsly in certain instances

1.2.3.4 rules have to be followed although student does not agree with it

1.2.3.5 benefit of the doubt

## 1.3 Definition includes (how they interpret it)

### 1.3.1 engaging

1.3.1.1 attendancy

1.3.1.2 school sees students as representatives in the community

1.3.1.3 being presentable

1.3.1.4 immature behavior

1.3.1.5 knowledge and skills

1.3.1.6 positive presence

1.3.1.7 timeliness

### 1.3.2 being honest

1.3.2.1 cheating

1.3.2.2 lying

1.3.2.3 plagiarism

1.3.2.4 not obeying rules and regulations of the school

1.3.2.5 illegal behavior outside the scool

1.3.2.6 misuse of alcohol

### 1.3.3 respect

1.3.3.1 inappropriate comments

1.3.3.2 involves behavior with social media

1.3.3.3 dress code

1.3.3.4 inclusivity

1.3.3.5 privacy violations

1.3.3.6 respect for faculty

1.3.3.7 respect for patients

1.3.3.8 respect for peers

1.3.3.9 disrespectful behavior about cadavers

1.3.3.10 not accusing others, but give benefit of the doubt

### 1.3.4 self-awareness

1.3.4.1 division between private views and professional behaviors

### 1.3.5 other

1.3.5.1 being able to identify unprofe..

1.3.5.2 ethical behavior

1.3.5.3 serves positive purpose

1.3.5.4 things that would eventually affect ..

1.3.5.5 you are the kind of colleague that we want to have in the future

## 1.4 Institutional response to unprofessional behaviour

1.4.1 institution offers remediation

1.4.2 Dean's letter

1.4.3 expel (with caveat)

1.4.4 second chance

1.4.5 students unprofessional behaviors are addressed very soon

1.4.6 anonymously reporting does not lead to interventions

## 1.5 Institutional education on professionalism

1.5.1 feels institution did a good job

1.5.1.1 faculty member to talk to anonymously

1.5.1.2 if student does not improves behavior after addressing personally, second step is report to faculty anonymously

1.5.1.3 institutional website to report peer students anonymously

1.5.1.4 clerkship coordinators ask for any problems

1.5.1.5 good office of professionalism

1.5.1.6 professionalism student council

### 1.5.2 not always taught in medical school to discuss unprofessional behavior

1.5.2.1 not enough education about professionalism issues

1.5.2.2 need to have more practical experience

1.5.2.3 trained how to report, but not enough

### 1.5.3 taught how to respond to unprofessional behavior yes

1.5.3.1 feedback sandwich

1.5.3.2 role modelling

1.5.3.3 talk to person first and report later if behavior not improves

1.5.3.4 comfort: address directly, discomfort: escalate

## 1.6 What should change to improve the situation

### 1.6.1 need for strengthening professionalism training

1.6.1.1 formal training in how to address UB

1.6.1.2 I want to learn how to give feedback in hierarchical situations

1.6.1.3 discussion professionalism dilemmas

1.6.1.4 helping student to understand what is expected from them

1.6.1.5 I would like to see more profe..

1.6.1.6 interact with different groups and overcome difficulties in the workplace

1.6.1.7 let students see the benefit of the professionalism subjects

1.6.1.8 possibility to speak to a peer in same hierarchical level

### 1.6.2 we need to be supportive to each other

### 1.6.3 students have to be part of policy making, make the change

### 1.6.4 task for faculty

1.6.4.1 critical feedback has to be given in time

1.6.4.2 faculty have to take responsibility

1.6.4.3 faculty have to maintain the rules if they have posed them upon students

1.6.4.4 faculty should be approachable and relatable

1.6.4.5 faculty should model the right behaviors themselves

1.6.4.6 mentor who you can trust

### 1.6.5 evaluating has to change

1.6.5.1 give grades for professionalism assignments

1.6.5.2 no summative evaluations

1.6.5.3 discussing unprofessionalism without punitive actions

### 1.6.6 structural change is needed

# 2. Faculty/resident

## 2.1 hesitating

2.1 hesitating to talk about unprofessional behavior even when the interview is anonymous

## 2.2 most faculty been professional

2.2 most faculty been professional

## 2.3 observed unprofessional behaviour among faculty

2.3.1 Yes

### 2.3.2 No

### 2.4 observed during…

### 2.4.1 lecture

### 2.4.2 one on one with attending

### 2.4.3 ward

### 2.4.4 exam

### 2.4.5 small group session

### 2.4.6 interaction with student council

### 2.4.7 interaction with administration

## 2.5 behaviors observed

### 2.5.1 failure to engage/lack of accountability

2.5.1.1 residents are often tired and weary

2.5.1.2 follow up on promises

2.5.1.3 results of exam not communicated in time

2.5.1.4 resident not sanitizing hands between patients

2.5.1.5 residents cutting corners

2.5.1.6 being late

2.5.1.7 exam did not represent study material

2.5.1.8 wrong information about grant application

2.5.1.9 faculty did not reach out to students to let them know that they had failed

2.5.1.10 no response on email

2.5.1.11 not promoting a good environment for learning

### 2.5.2 dishonesty

2.5.2.1 misrepresentation of facts

2.5.2.2 faculty incorrectly overruled student council

### 2.5.3 disrespectful behavior

2.5.3.1 faculty disrespectful to students

2.5.3.1.1 inappropriate behavior towards student

2.5.3.1.2 lack of value for student time

2.5.3.1.3 offensive remarks to students

2.5.3.1.4 texting and calling a student

2.5.3.1.5 treating students like children

2.5.3.1.6 express strong opinions which would not be the opinion of the student

2.5.3.1.7 faculty loses his temper

2.5.3.2 faculty disrespectful to patients

2.5.3.2.1 faculty had complaints about patien..

2.5.3.2.2 impose own views on patient

2.5.3.2.3 inappropriate remarks about patients

2.5.3.3 faculty disrespectful to faculty

2.5.3.3.1 faculty complaining about the school that I go to

2.5.3.3.2 speaking not politely about other faculty

2.5.3.3.3 yelling at colleagues

2.5.3.4 faculty biased views

2.5.3.4.1 inappropriate interacting with student from different sekse

2.5.3.4.2 sexist remarks

2.5.3.4.3 social injustice

### 2.5.4 faculty poor self-awareness

2.5.4.1 lack of openness to feedback

2.5.4.2 lack of value

2.5.4.3 not acknowledging students contribution

2.5.4.4 not acknowledging mistake

2.5.4.5 share too much personal information with the students

## 2.6 Causes of behaviour (as interpreted by student)

### 2.6.1 mistake

### 2.6.2 not acknowledging because of fear for legal issues

### 2.6.3 poor organization

### 2.6.4 individual personalities

### 2.6.5 it is not my fault but yours

### 2.6.6 stress of the job

### 2.6.7 others treated the attending wrong

### 2.6.8 difficult patient

## 2.7 Who displayed/context of behaviour

### 2.7.1 attending

### 2.7.2 facilitator in small group

### 2.7.3 midwife

### 2.7.4 resident

## 2.8 Intervened yes/no

### 2.8.1 no, not intervened

### 2.8.1 yes, intervened

2.8.1.1 students underreport unprofessional behaviors of attendings

## 2.9 Response

### 2.9.1 respond in the moment

### 2.9.2 responding after the moment

### 2.9.3 individual action

### 2.9.4 collective action

### 2.9.5 ignored

2.9.5.1 gossiping about faculty

2.9.5.2 nonverbal contact between students

2.9.5.3 not endorsed

2.9.5.4 students warn each other for behaviors of certain attendings

2.9.5.5 talked to mentor

2.9.5.6 try to deflect

2.9.5.7 completing of evaluations

### 2.9.6 intervened

2.9.6.1 strategic discussion with perpetrator

2.9.6.2 addressing in an inappropriate way, and apologize afterwards

2.9.6.3 mom took care of it by approaching a friend of the perpetrator and asking him to address it

2.9.6.4 response to professor was muted

2.9.6.5 students try to keep the UB small and de-escalate it as much as possible

### 2.9.7 report to higher level

2.9.7.1 reporting anonymously

2.9.7.2 reported to student government, which conveyed to faculty

### 2.9.8 intervened as representative of students

### 2.9.9 alternative action (not executed)

### 2.9.10 discussion among students

## 2.10 Reasons to respond/not respond

### 2.10.1 costs high

2.10.1.1 be seen as troublemaker or whiner

2.10.1.2 does not want to bring resident into trouble

2.10.1.3 fear of retaliation

2.10.1.4 fear of retaliation (grades)

2.10.1.5 internal struggle

2.10.1.6 it gets harder to address unprofessional behaviors as you go on

2.10.1.7 relations could be affected

### 2.10.2 costs low

2.10.2.1 I do not have to work with this person again

2.10.2.2 power in numbers made it easier to address the behavior

2.10.2.3 small group made it easier, more comfortable

### 2.10.3 positive value for himself or other students

2.10.3.1 express common feelings

2.10.3.2 making aware of behavior

2.10.3.3 patient should not suffer

2.10.3.4 personally affected

2.10.3.5 responsibility for your classmates

### 2.10.4 no value

2.10.4.1 only one time occurrence

2.10.4.2 personally not affected

2.10.4.3 we had not been hurt

2.10.4.4 I will not go in this specialty

2.10.4.5 I was only with that provider for 8 hours

2.10.4.6 I built up a tolerance for unprofessionalism

2.10.4.7 cheating is the only way of surviving

### 2.10.5 expectancy of success is high

2.10.5.1 because you knew the facilitator for a longer time

2.10.5.2 I am realistic and practical

2.10.5.3 clerkship director approachable

2.10.5.4 good relationship with dean makes it easier to report

2.10.5.5 I'm used to speaking up, so it..

2.10.5.6 I think it's even more product..

2.10.5.7 investment

2.10.5.8 there's a potential for us to change

### 2.10.6 nothing would result, expectancy low

2.10.6.1 afraid to be biased

2.10.6.2 dependent

2.10.6.3 did not know how to respond

2.10.6.4 especially when they're older ..

2.10.6.5 felt powerless

2.10.6.6 hierarchy

2.10.6.7 in clinical years you are pretty separated from your peers which makes a common response more difficult

2.10.6.8 long time ago

2.10.6.9 not engaged with the system yet

2.10.6.10 not feeling comfortable saying anything

2.10.6.11 perpetrator not approachable

2.10.6.12 waste of resources

2.10.6.13 what benefit would this have fo..

2.10.6.14 you don't know who they are, o..

## 2.11 Outcome of intervention

### 2.11.1 facilitator was taken off the job temporarily

### 2.11.2 perpetrator tried to pin blame on student

### 2.11.3 apologies

2.11.3.1 apologized

2.11.3.2 clerkship director apologized on behalf of attending

### 2.11.4 nothing changed

2.11.4.1 after talking about it everyone just got over it

2.11.4.2 I managed to brush it off

2.11.4.3 we got over it

### 2.11.5 changes were made for future students

2.11.5.1 clerkship director talks to attendings en residents

2.11.5.2 discussed in professionalism Council of hospital

2.11.5.3 facilitator got additional training

2.11.5.4 facilitator improved behavior

### 2.11.6 unsatisfactory outcome

2.11.6.1 explanation, but not satisfying

2.11.6.2 no action from faculty followed

2.11.6.3 no apologies

2.11.6.4 no measures taken to avoid repetition

2.11.6.5 perpetrator said he got it, but students do not know if he really did

2.11.6.6 student still avoids meeting the perpetrator

2.11.6.7 students were brushed off

2.11.6.8 the institution did not address unprofessional behavior of faculty

### 2.11.7 felt good that it was taken care off

# 3. Fellow student

## 3.1 Hesitating

3.1 hesitating to speak about unprofessional behaviror

## 3.2 most peers been professional

3.2 most peers been professional

## 3.3 observed unprofessional behavior among students (yes/no)

3.3 YES observed unprofessional behavior among students

## 3.4 observed during…

### 3.4.1 clerkship

### 3.4.2 Facebook post was taken down but screenshot still on the Internet

## 3.5 behaviors observed

### 3.5.1 failure to engage

3.5.1.1 being absent

3.5.1.2 cutting corners

3.5.1.3 leaving too early

3.5.1.4 let others do the work

3.5.1.5 not productive

3.5.1.6 timeliness

### 3.5.2 dishonesty

3.5.2.1 "steal" patient from other student

3.5.2.2 lying

3.5.2.3 cheating on exams

3.5.2.4 give false excuses for absence

3.5.2.5 plagiarizing

3.5.2.6 not keeping to rules of school

### 3.5.3 disrespect

3.5.3.1 students disrespectful to faculty

3.5.3.2 student disrespect to peers

3.5.3.2.1 abrasive behavior to peers

3.5.3.2.2 competitiveness among students

3.5.3.2.3 dismissive of the underclassmen..

3.5.3.2.4 making inappropriate comments about peers

3.5.3.3 disrespect to patients

3.5.3.3.1 speaking about patients in a disrespectful way

3.5.3.4 students' disrespectful behavior regarding cadavers

3.5.3.5 inclusivity

3.5.3.5.1 justice and equity pieces

3.5.3.5.2 racism, sexism

3.5.3.5.3 unfair

3.5.3.6 social media

3.5.3.6.1 even in closed social media groups you should be careful, anyone could make a screenshot and it could go public certainly

3.5.3.7 privacy violations

### 3.5.4 poor self-awareness

3.5.4.1 not open to others' views

## 3.6 cause of behaviour (as interpreted by student)

### 3.6.1 new to the system

### 3.6.2 passionate about topic

### 3.6.3 type A behavior

### 3.6.4 student is not necessary for the job

### 3.6.5 stress of the job

## 3.7 who displayed /context of behaviour

### 3.7.1 peer on social media

### 3.7.2 one student on student council

## 3.8 intervened yes/no

3.8.1 no

3.8.1.1 not being able to address it was very difficult

### 3.8.2 responded yes

## 3.9 intervention

### 3.9.1 respond in the moment

### 3.9.2 responding after the moment

3.9.2.1 I might do that after I graduated

### 3.9.3 individual action

### 3.9.4 collective action

### 3.9.5 ignore

3.9.5.1 backing up fellow student

3.9.5.2 focusing on the future instead of the incident

3.9.5.3 gossiping about other students

3.9.5.4 I was a bystander

3.9.5.5 less likely to help this person

3.9.5.6 try to block it out

### 3.9.6 intervened

3.9.6.1strategic discussion with perpetrator

3.9.6.2 find someone who knows the perpetrator well, so that he can address the behavior

3.9.6.3 counterproductive to what other groups were trying to do

3.9.6.4 bring it up as like a joke

3.9.6.5 after personal reflection on how to react appropriately

### 3.9.7 reporting to higher level

3.9.7.1 reporting to higher level, anonymously

3.9.7.2 formal complaint to student council on behalf of group

### 3.9.8 social media post to address perpetrator

### 3.9.9 take action as student representative

3.9.9.1 alter bylaws for student council

3.9.9.2 remove from student council

### 3.9.10 discussion among students

### 3.9.11 problem escalated only when required

3.9.11.1 students try to keep the UB small and de-escalate it much as possible

### 3.9.12 alternative action (not executed)

### 3.9.13 completion of evaluations

## 3.10 reason for taking action/no action

### 3.10.1 costs are high

3.10.1.1 be seen as troublemaker or whiner

3.10.1.2 does not want to bring other student into trouble

3.10.1.3 does not want to lose face

3.10.1.4 don't want to be identified as..

3.10.1.5 don't want to hurt each other'..

3.10.1.6 fear of damaging relations

3.10.1.7 fear of retaliation

3.10.1.8 not rock the boat

3.10.1.9 prevent peer from getting into trouble

3.10.1.10 responding makes you nervous

3.10.1.11 too busy

3.10.1.12 You don't want to be the tattl..

3.10.1.13 you want to fit in the group

### 3.10.2 costs low

3.10.2.1 being a friend makes it easier to respond individually

3.10.2.2collective action makes it easier to address UB

3.10.2.3 easier to address a peer than to address faculty

3.10.2.4 not punitive

3.10.2.5 you can report, and honor council takes care of it

### 3.10.3 value high

3.10.3.1 action was fair

3.10.3.2 collective responsibility, we should protect each other

3.10.3.3 curious about reason for behavior

3.10.3.4 helping student to understand what is expected from them

3.10.3.5 patients should not suffer

3.10.3.6 personally affected

3.10.3.7 social contract

3.10.3.8 understanding the reason for the unprofessional behavior

3.10.3.9 value for himself and other students

3.10.3.10 value for other students

3.10.3.11 we have to do that as physicians, so we have to learn it now

### 3.10.4 no value

3.10.4.1 the patient wasn't getting hur..

3.10.4.2 the student wasn't necessarily..

3.10.4.3 it is being handled

### 3.10.5 expectancy of success is high

3.10.5.1 because I'm the type of person..

3.10.5.2 culture of feedback giving among teachers and students

3.10.5.3 I have a good relationship..

3.10.5.4 I knew the perpetrator kind of well

3.10.5.5 important to know someone on the honor council

3.10.5.6 small class

### 3.10.6 nothing would result, expectancy low

3.10.6.1 too soon after event

3.10.6.2 shy, being non-confrontational

3.10.6.3 perpetrator not open for feedback

3.10.6.4 defensive reaction

3.10.6.5 difficult communicating with other students because they were angry

3.10.6.6 council bylaws did not allow that

3.10.6.7 does not know how to respond

3.10.6.8 location was not suitable for action

3.10.6.9 I didn't know that person as w..

## 3.11 Outcome of intervention

### 3.11.1 perpetrator upset

### 3.11.2 others appreciated and confirmed the intervention

### 3.11.3 apologetic

### 3.11.4 student representative between two fires

### 3.11.5 changes were made

3.11.5.1 behavior will not change, but he will not do it when I am around

3.11.5.2 perpetrator stopped

3.11.5.3 perpetrator took post down from Facebook right away

3.11.5.4 set the tone for whole group

3.11.5.5 slightly improved behavior

### 3.11.6 unsatisfactory outcome

3.11.6.1 behavior did not improve

3.11.6.2 faculty overruled the student professionalism council

3.11.6.3 perpetrator did not accept feedback immediately

3.11.6.4 students move apart

3.11.6.5 tension between faculty and student council

### 3.11.7 perpetrator listened to feedback

### 3.11.8 sanction

3.11.8.1 he actually got a professionalism violation ..

3.11.8.2 student council recommended a sanction

### 3.11.9 he will get another chance

### 3.11.10 made me feel bad

### 3.11.11 So it definitely made me lose ..

### 3.11.12 felt good

# 4. Own unprofessional behaviour

## 4.1 acted unprofessional

### 4.1.1 no

### 4.1.2 yes

## 4.2 behavior mentioned

### 4.2.1 failure to engage

4.2.1.1 being absent

4.2.1.2 cutting corners

4.2.1.3 not following up request from midwife

4.2.1.4 timeliness

### 4.2.2 dishonesty

### 4.2.3 disrespect

4.2.3.1 being emotional with patient

4.2.3.2 being judgmental about patient

4.2.3.3 communication too aggressive

4.2.3.4 inappropriate remark about patient

4.2.3.5 privacy violation

4.2.3.6 unprofessional behavior on social media

### 4.2.4 poor self-awareness

## 4.3 received response/feedback

### 4.3.1 peer gave feedback

4.3.1.1peer made me aware of my judgmental behavior

4.3.1.2 peer student said I was too harsh

### 4.3.2 somebody reported it to a higher level

4.3.2.1 peer reported my behavior

4.3.2.2 would be better if the person had come to me personally

### 4.3.3 conversation with attending

### 4.3.4 conversation with dean

### 4.3.5 addressing whole group instead of me personally

### 4.3.6 I realized it myself before peers could respond

## 4.4 resulting in

### 4.4.1 crying

### 4.4.2 feed forward learning goals myself

### 4.4.3 feedback made me formulate an apology

### 4.4.4 feedback not effective if personal issues are in the way

### 4.4.5 found it intimidating

### 4.4.6 getting accustomed to receiving feedback

### 4.4.7 I listened to feedback and tried to improve behavior

### 4.4.8 my peers have helped me become more professional

### 4.4.9 they were right to pull me in

### 4.4.10 try to verify the feedback with others

### 4.4.11 you are not willing to accept feedback from a person who behaves unprofessionally himself

## 4.5 reason for own unprofessional behavior

### 4.5.1 I did not find that in the interest of the patient

### 4.6.2 no one would care about my absence

# 5. Student characteristics

5.1 student representative

5.2 Second year student

5.3 third year student

5.4 4th year student
